# The Prediction of Intrapartum Fetal Compromise According to the Expected Fetal Weight

**DOI:** 10.3390/jpm15040140

**Published:** 2025-04-01

**Authors:** José Morales-Roselló, Alicia Martínez-Varea, Blanca Novillo-Del Álamo, Carmen Sánchez-Arco, Asma Khalil

**Affiliations:** 1 Department of Obstetrics and Gynaecology, La Fe University and Polytechnic Hospital, Avenida Fernando Abril Martorell 106, 46026 Valencia, Spain; martinez_alivar@gva.es (A.M.-V.); novillo_bla@gva.es (B.N.-D.Á.); sanchez_cararc@gva.es (C.S.-A.); 2 Department of Pediatrics, Obstetrics and Gynecology, Faculty of Medicine, University of Valencia, 46010 Valencia, Spain; 3 Fetal Medicine Unit, St George’s Hospital, St George’s University of London, Blackshaw Road, London SW170QT, UK; asma.khalil@stgeorges.nhs.uk

**Keywords:** estimated fetal weight, cerebroplacental ratio, middle cerebral artery, intrapartum fetal compromise, fetal growth restriction, genetic growth potential

## Abstract

**Objectives:** To assess the predictive accuracy of the expected fetal weight in the third trimester (ExFW3t), based on the estimated fetal weight (EFW) at mid-trimester ultrasound scan, for the prediction of intrapartum fetal compromise (IFC) (an abnormal intrapartum fetal heart rate or intrapartum fetal scalp pH requiring urgent cesarean section). **Methods:** This retrospective study included 777 singleton pregnancies that underwent a 20-week study and a 3t scan. The extrapolated EFW at 20 weeks to the 3t or ExFW3t was considered a proxy of the potential growth. The percentage difference with the actual 3t EFW (%ExFW3t) was compared with other ultrasonographic and clinical parameters—EFW centile (EFWc), middle cerebral artery pulsatility index (MCA PI) in multiples of the median (MoM), umbilical artery (UA) PI MoM, cerebroplacental ratio (CPR) MoM, and maternal height—for the prediction of IFC by means of the area under the curve (AUC) and Akaike Information Criteria (AIC). **Results:** Pregnancies with IFC presented higher values of UA PI MoM (1.19 vs. 1.09, *p* = 0.0460) and lower values of population and Intergrowth EFWc (45.9 vs. 28.9, *p* < 0.0001, 48.4 vs. 33.6, *p* = 0.0004), MCA PI MoM (0.97 vs. 0.81, *p* < 0.0001), CPR MoM (1.01 vs. 0.79, *p* < 0.0001), %ExFW3t (89.9% vs. 97.5%, *p* = 0.0003), and maternal height (160.2 vs. 162.9, *p* = 0.0083). Univariable analysis selected maternal height, EFWc, %ExFW3t, and UA PI MoM as significant parameters. However, %ExFW3t did not surpass the prediction ability of cerebral Doppler. Finally, multivariable analysis showed that the best models for the prediction of IFC resulted from the combination of cerebral Doppler (MCA PI MoM or CPR MoM), fetal weight (%ExFW3t or EFWc), and maternal height (AUC 0.75/0.76, AIC 345, *p* < 0.0001). **Conclusions:** Fetal weight-related parameters, including %ExFW3t, a proxy of the proportion of potential growth achieved in the 3t, were less effective than fetal cerebral Doppler for the prediction of IFC. The best performance was achieved by combining hemodynamic, ponderal, and clinical data.

## 1. Introduction

One of the primary goals of obstetric care is to predict intrapartum fetal compromise (IFC) (intrapartum loss of fetal welfare) using a quick, accessible, and accurate method. Until recently, the prognosis relied on the estimated fetal weight centile (EFW), as the focus of fetal well-being was primarily on ponderal growth [1]. However, the EFW has been surpassed by the cerebroplacental ratio (CPR), which demonstrates greater accuracy in predicting IFC [2].

Fetal growth restriction (FGR) is defined as the inability to achieve the fetal growth potential (GP). Unfortunately, direct methods to measure this growth are not available. One approach to measuring it involves customizing growth expectations based on factors such as maternal ethnicity, height, weight, and parity [3]. Alternatively, a second possibility would be to consider that most mechanisms influencing GP are of placental origin and exert their effect mainly in the second half of pregnancy. Accordingly, EFW at mid-pregnancy may serve as a proxy for GP, with its extrapolation to the second half of pregnancy providing an estimate of the growth the fetus might achieve in the absence of external influences [4].

Following this rationale, and based on the EFW at 20 weeks, we calculated the percentage of the expected weight achieved in the third trimester (3t) and evaluated whether this new ponderal parameter outperformed other predictive measures, including EFW centile (EFWc) (local and Intergrowth 21st), middle cerebral artery pulsatility index (MCA PI) multiples of the median (MoM), umbilical artery (UA) PI MoM, cerebroplacental ratio (CPR) multiples of the median (MoM), and clinical data for the prediction of IFC.

## 2. Materials and Methods

This was a retrospective study of 777 singleton pregnancies with accurate gestational ages (GA) according to the 12 weeks’ crown-rump length (CRL), attending the ultrasound unit of La Fe Hospital, Valencia, Spain, that underwent a mid-pregnancy (20 weeks) plus a 3t scan and were subsequently delivered within the following two weeks after induction or spontaneous onset of labor. Mid-pregnancy ultrasound was performed at week 20 ± 1, and included biparietal diameter (BPD), head circumference (HC), abdominal circumference (AC), and femur length (FL).

Third-trimester ultrasound was performed between 30 + 0 and 40 + 6 weeks and included the same parameters plus a Doppler evaluation of the UA PI, MCA PI, and CPR, representing them as the determinants with the highest accuracy in predicting IFC [2].

EFW at the mid-trimester and the 3t scan was calculated according to the Hadlock 4 formula and transformed, for comparison purposes, into local and Intergrowth 21st EFWc [5]. For the same reason, MCA PI, UA PI, and CPR values were converted into MoM, dividing each value by the 50th centile (median) at each GA, as described earlier [6,7]. The UA and MCA were recorded using color and pulse Doppler according to standard protocols [6,7], and the CPR was calculated as the simple ratio between the MCA PI and the UA PI [6,8]. Ultrasound assessment was performed using General Electric Voluson^®^ (E8/E6/S8/730) ultrasound machines, with 2–8 MHz convex probes, during fetal quiescence, in the absence of fetal tachycardia and keeping the insonation angle with the examined vessels as small as possible. Only one examination per fetus (the last) was included.

### 2.1. Rationale to Calculate the Growth Potential

The cornerstone and main rationale of this work was to consider, as mentioned previously, that EFW at mid-pregnancy (20 weeks) might reflect a proxy of the GP prior to the action of later influences [9]. Accordingly, if this EFW was extrapolated to the 3t [4], differences with the actual EFW might represent the percentage of the potential or expected weight achieved.

### 2.2. Formulas to Calculate the Percentage of Expected Weight

This percentage was calculated with an extrapolation procedure that applied MoM according to the following formulas:

EFW Median = (−3.266164164 + (0.368135209 ∗ GA) − (0.006318278 ∗ GA2))

where GA is the gestational age expressed in decimals (33.14 for 33 weeks plus 1 day, 33.29 for 33 weeks plus 2 days, and so on).

EFW MoM 20 weeks = EFW 20 weeks/EFW median 20 weeks.

Expected weight at the 3rd trimester (ExFW3t) = EFW MoM 20 w ∗ EFW median 3t

Percentage of ExFW at the 3rd trimester (%ExFW3t) = (100 + ((EFW3t − ExFW3t)/ExFW3t) ∗ 100).

Accordingly, a fetus who in the 3t scan grew 25% more than its ExFW3t presented a %ExFW3t of 125%, while a fetus who grew 25% less than the ExFW3t presented a %ExFW3t of 75%.

This percentage was evaluated afterward and compared with other ultrasonographical variables (EFWc, MCA PI MoM, UA PI MoM, and CPR MoM) and clinical data for the prediction of IFC. Multiple pregnancies and those complicated by major fetal abnormalities or aneuploidies were excluded. Only fetuses undergoing induction or spontaneous onset of labor were included in the study. These fetuses were managed according to the local protocol [10], although the managing physicians were not blinded to the fetal biometry or Doppler values.

Outcome data, including birthweight, mode of delivery, Apgar score, and cord arterial pH, were collected after birth. IFC was defined in case of abnormal intrapartum fetal heart rate [11] or intrapartum fetal scalp pH [12] requiring urgent cesarean section, which is performed in all cases of suspicious CTG with sufficient access to the fetal head. However, IFC was not considered in case of urgent instrumental vaginal delivery. Other data variables included maternal age, pre-pregnancy weight, height, body mass index, parity, and number of gestations, plus GA at examination and delivery (in weeks), interval between ultrasound assessment and birth, EFW, EFWc, BW, BW centile (BWc), UA PI MoM, MCA PI MoM, CPR MoM, fetal gender, type of labor onset (induction and spontaneous), mode of delivery (assisted or spontaneous vaginal delivery and cesarean section due to failure to progress or IFC), Apgar scores at 5 min, and neonatal cord arterial pH and neonatal outcomes (transfer to the maternal and neonatal wards or neonatal intensive care unit, NICU).

Continuous variables were analyzed using the median and interquartile range (IQR), while categorical variables were analyzed as numbers and percentages.

Univariable logistic regression analysis, including the odds ratios (OR), their 95% confidence intervals (CI), β-coefficients, and their *p*-values, was used in every studied parameter to determine its importance. Afterward, multivariable logistic regression analysis was applied to identify and adjust for potential confounders in the explanation of IFC. The predictive accuracy of all the parameters and models was evaluated and compared using the Akaike information criteria (AIC) and ROC curves analysis, with the detection rate (DR), false positive rate (FPR), and area under the curve (AUC). The best models were those with the lowest AIC and the highest AUC, while significant differences were represented by a difference of 2 units in the AIC.

Comparisons of the continuous data variables were made using the Mann–Whitney test, while the Chi-Square test was used to compare binary or categorical data variables. Statistical analysis was performed with StatPlus^®^ for Mac, version 7, and GraphPad Prism^®^ for Mac, version 5. Significance was established at *p* values < 0.05.

## 3. Results

The study population is described in Table 1. In summary, the mean maternal age, pre-pregnancy weight, height, body mass index (BMI), and GA at the 3t examination and delivery were 32.6 years, 62.3 kg, 162.7 cm, 20.4 kg/m^2^, 39, and 39.9 weeks, respectively. Moreover, there were equal numbers of male and female fetuses; 14% of women smoked, and 52.9% were nulliparous. Concerning labor, half of the pregnancies were induced, with spontaneous and uneventful deliveries (59.1%), and only 6.2% had an emergency cesarean section for IFC. The frequency of Apgar scores below 7 at 5 min and pH < 7.10 was 0.5% and 2.3%, respectively.

**Figure 1:**
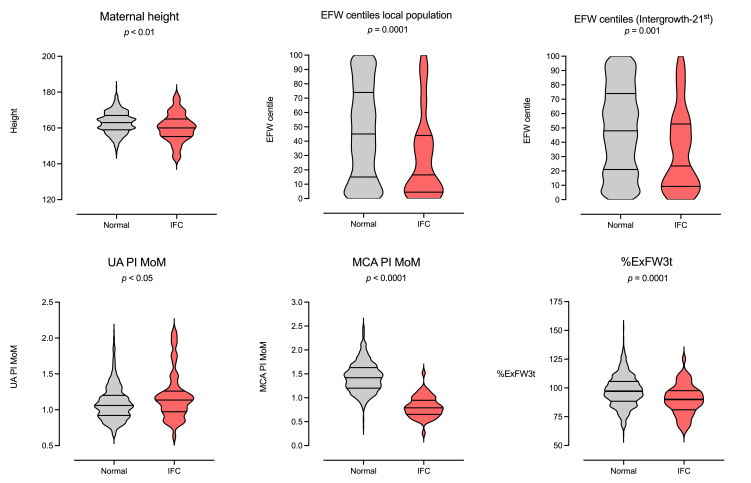
Violin plots with the promising parameters for the prediction of intrapartum fetal compromise.

Table 1 also shows the differences between fetuses experiencing IFC and those that did not, while Figure 1 shows the prediction plots of the promising parameters that presented differences. Fetuses experiencing IFC had significantly shorter mothers, a shorter examination–delivery interval, lower population and Intergrowth EFWc, lower BW and BWc, lower MCA PI MoM and CPR MoM, higher UA PI MoM, higher frequency of male sex, and a lower %ExFW3t. Finally, concerning delivery, they had significantly more inductions of labor, lower arterial pH < 7.10, and a higher rate of admission to the neonatal ward. Interestingly, there were no significant differences either in the 20-week EFW, in the 20-week EFW MoM, or in the ExFW3t, proving that both study groups were similar with similar GPs.

**Figure 2:**
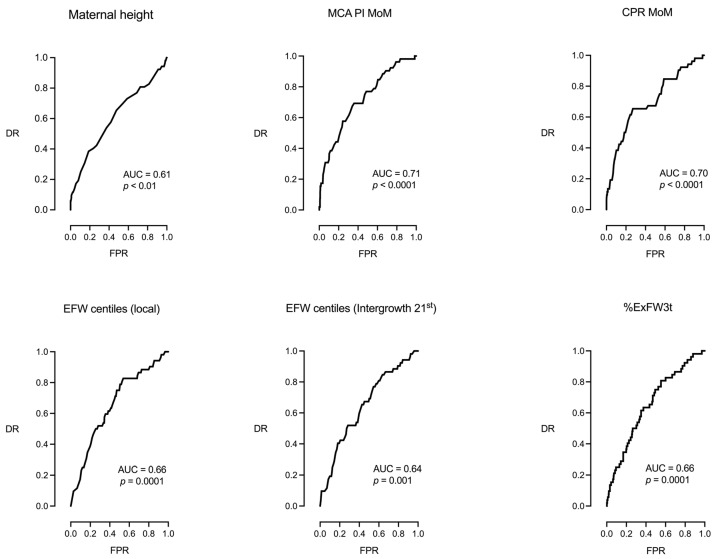
ROC curves of the parameters presented in Table 2 that showed statistical significance.

Table 2 shows the univariable logistic regression analysis for the prediction of IFC, while Figure 2 plots the ROC curves of parameters that presented statistical significance in this analysis. Maternal height was the only clinical parameter that predicted the risk of IFC. Regarding fetal parameters, the MCA PI MoM and the CPR MoM were the most important parameters, although the UA PI MoM, the EFW local population and intergrowth 21st centiles, and the %ExFW3t were also predictive.

**Figure 3:**
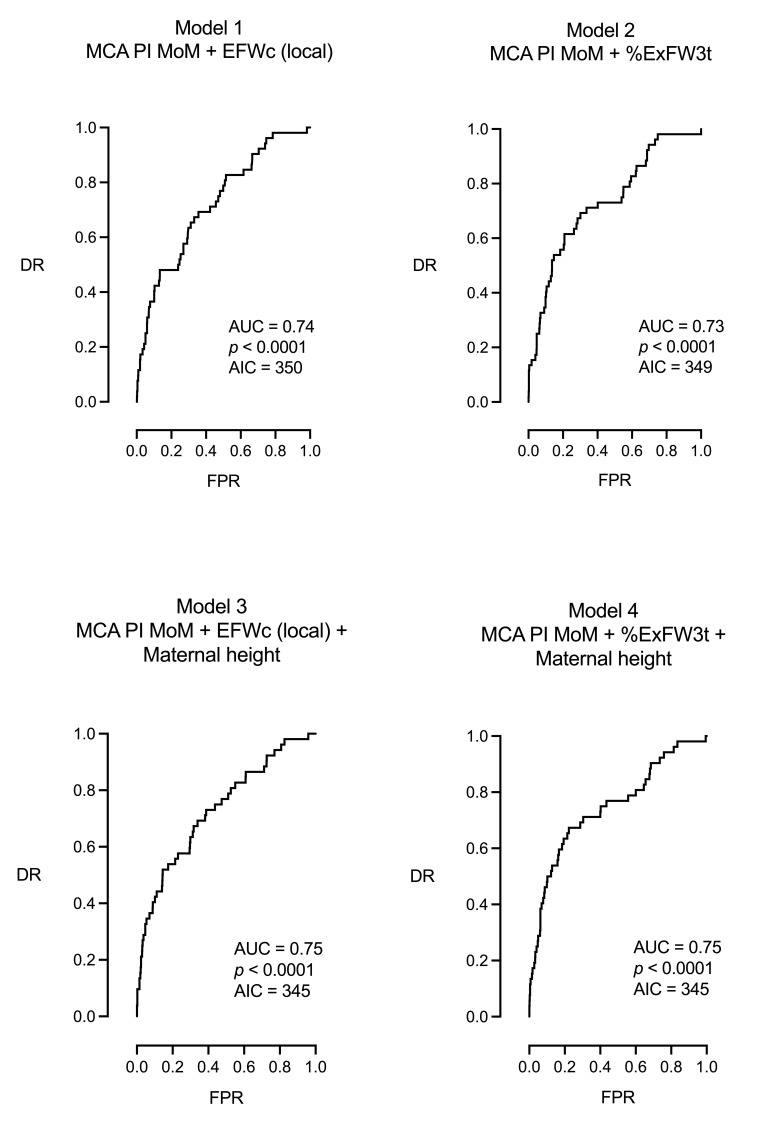
ROC curves of the multivariable models presented in Table 3.

**Figure 4:**
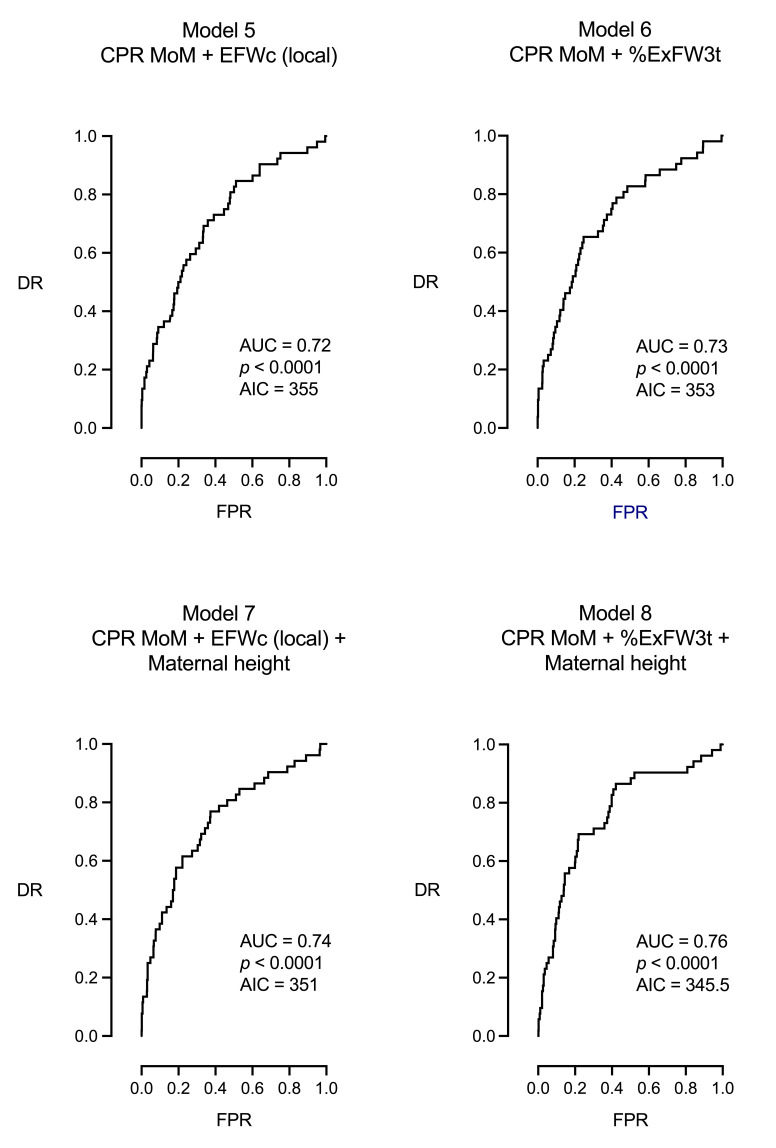
ROC curves of the multivariable models presented in Table 4.

The significant parameters were subsequently combined into eight models, which always included either the MCA PI MoM (Table 3 and Figure 3) or the CPR MoM (Table 4 and Figure 4), being the parameters with the highest predictive accuracy. Concerning MCA PI MoM models, model 1 combined MCA PI MoM with EFW local centiles, model 2 combined MCA PI MoM with %ExFW3t, and models 3 and 4 combined models 1 and 2 with maternal height, representing the best models for the prediction of IFC (both AUC = 0.75, AIC = 345, *p* < 0.0001).

Concerning CPR MoM models, model 5 combined CPR MoM with local EFW centiles, model 6 combined CPR MoM with %ExFW3t, model 7 combined model 5 with maternal height, and finally, model 8 combined model 6 with maternal height, representing the best model for the prediction of IFC (AUC = 0.76, AIC = 345.5, *p* < 0.0001). A summary of the models’ performance, ordered according to the lowest AIC (highest accuracy and reproducibility), is shown in Table 5.

## 4. Discussion

Considering that mid-pregnancy EFW might be interpreted as a proxy of potential growth and knowing the actual 3t EFW, it was possible to calculate the ExFW and the percentage of ExFW at the time of the 3t scan (%ExFW3t). Unfortunately, regarding the prediction of IFC, this parameter performed like other weight-centile variants: it had lower predictive accuracy than the cerebral Doppler. Finally, the best prediction was achieved by combining hemodynamic, ponderal, and clinical data.

Rossavik et al. and Deter et al. [9,13] were the first authors to describe the concept of “expected measurement” in the 3t using measurements obtained in the first half of pregnancy. These authors calculated a proxy of growth potential (the “expected value”) and the “growth potential realization index”, which was the measurement at birth divided by the expected value at this GA. Unfortunately, despite their extremely meritorious work, the complexity of their mathematical calculation made it rather difficult to use their methodology in routine clinical practice. Subsequently, a few authors applied the rationale of using fetal growth in the first part of pregnancy to calculate a proxy of potential growth [14]. Santonja-Lucas et al. [15] described a similar and simpler procedure, and, subsequently, Morales-Roselló, with this method, showed the possibility of obtaining predicted growths and comparing them with the actual birth weight [4,16], finally proving that the worse outcomes were related to the highest differences between them [17,18].

Although the methodology of the latter studies, which calculated the potential growth using measurements taken in the first half of pregnancy, was less complicated than the method of Rossavik and Deter [9,13], it was still challenging to use in clinical practice. Therefore, in the current work, we strived to use a simpler method with MoM, a way to extrapolate mid-pregnancy growth to the second part of pregnancy based on the medians or 50th centiles of the local population. This rationale considered, as in the previous methodologies, that the EFW at this early GA would reflect the potential growth prior to the action of the factor influencing growth during the second half of pregnancy.

An interesting demonstration of the plausibility of this approach was that normal fetuses and fetuses presenting IFC, with different %ExFW3t, presented similar EFW at 20 weeks and similar ExFW3t. This proved that only the presence of different %ExFW3tcaused the differences in IFC at birth.

Our work concluded that the EFW, in all its expressions, performed poorer than fetal brain hemodynamics to predict IFC. This is in line with current research, which describes, for the EFW, low performance in predicting adverse outcomes [19,20,21]. Interestingly, this ability gets lower as labor approaches [22], contrary to that of cerebral Doppler, which increases with shorter intervals to labor, making it ideal for short-term predictions of IFC [23,24].

Our results show that using the EFW of the 20-week ultrasound, we can extrapolate a proxy of the fetal GP and calculate the proportion of the genetic growth finally achieved by the fetus. Unfortunately, the performance of this method, which personalizes growth surveillance to individual standards, remains below that of both cerebral Doppler measurements, MCA PI and CPR. In this regard, despite fetal cerebral Doppler surpassing fetal biometry for the prediction of IFC, the best prediction is still achieved with a combination of hemodynamic, ponderal, and clinical parameters. Therefore, a combination of these examinations is mandatory, but always bearing in mind the superior importance of the cerebral flow examination [25].

The strengths of the study are its novelty, as this is the first study to evaluate IFC by means of an easily calculated proxy of potential growth, and the statistical methodology, using logistic regression analysis combined with ROC curves and AIC to evaluate models to predict IFC. Conversely, limitations include the retrospective nature, the absence of other parameters like the uterine artery Doppler, and the possibility of intervention bias, as the managing physicians were not completely blinded to the Doppler examination results. Future studies will be needed to evaluate this parameter prospectively and to compare it with the third-trimester evaluation of the uterine Doppler.

## 5. Conclusions

All fetal weight-related parameters, including %ExFW, a proxy for the potential growth achieved in the 3t, are less effective than cerebral Doppler for the prediction of IFC. However, the most accurate prediction of IFC is achieved through a combination of hemodynamic, ponderal, and clinical data.

## Figures and Tables

**Table 1:** Description of the study population (N = 777).

Parameter	All (N = 777)	No IFC (N = 725)	IFC (N = 52)	2 vs. 3 *
	**Mean (SD); Median (IQR)**	**Mean (SD); Median (IQR)**	**Mean (SD); Median (IQR)**	***p*** **-value**
Maternal age in years	32.6 (5.1); 33 (29, 36)	32.5 (5.2); 33 (29, 36)	33.6 (4.3); 34 (31, 36)	1.0000
Maternal pre-pregnancy weight (kgs)	62.3 (11.7); 60 (55, 67)	62.3 (11.7); 60 (55, 67)	63 (12.6); 60 (54.2, 69.5)	0.7619
Maternal height (cm)	162.7 (6.2); 163 (159, 167)	162.9 (6.0); 163 (159, 167)	160.2 (7.8); 160 (155, 165)	0.0083
Maternal body mass index, Kg/m^2^	20.43 (0.5); 23 (21, 25)	23.4 (4.1); 23 (21, 25)	24.5 (5); 23 (22, 27.7)	0.1487
Gestational age at 3rd trim. scan (weeks)	39 (1.3); 39.3 (38.1, 40)	39 (1.2); 39.3 (38.3, 40)	38.5 (2.1); 39.1 (37.6, 40)	0.6245
Gestational age at delivery (weeks)	39.9 (1.28); 40.1 (39.4, 40.9)	39.9 (1.2); 40.1 (39.4, 40.9)	39.2 (2.2); 40.1 (38.3, 40.7)	0.1380
EFW (Hadlock), 20 weeks (grams)	362.3 (53.2); 357 (329, 390)	362.6 (53.84); 357 (329, 390.5)	358.2 (43.14); 357 (335.5, 378.5)	0.8464
EFW MoM, 20 weeks	0.97 (0.1); 0.96 (0.90, 1.03)	0.97 (0.11); 0.96 (0.90, 1.03)	0.96 (0.1); 0.97 (0.90, 1.03)	0.9834
UA PI MoM, 3rd trim.	1.10 (0.25); 1.06 (0.92, 1.21)	1.09 (0.24); 1.06 (0.92, 1.20)	1.19 (0.33); 1.13 (0.97, 1.26)	0.0460
MCA PI MoM, 3rd trim.	0.97 (0.22); 0.95 (0.80, 1.12)	0.97 (0.22); 0.96 (0.82, 1.13)	0.81 (0.21); 0.79 (0.65, 0.94)	<0.0001
CPR MoM, 3rd trim.	0.99 (0.29); 0.99 (0.78, 1.19)	1.01 (0.29); 0.99 (0.80, 1.20)	0.79 (0.30); 0.60 (0.75, 1.03)	<0.0001
EFW (Hadlock), 3rd trim. (grams)	3135 (532); 3166 (2847, 3498)	3157 (518); 3182 (2874, 3512)	2828 (624); 2908 (2420, 3346)	1.0000
EFW centile (local), 3rd trim.	44.8 (32); 43 (14, 72.5)	45.9 (31.9); 45 (15, 74)	28.9 (29.2); 16.5 (4.5, 44)	<0.0001
EFW centile (Intergrowth-21st), 3rd trim.	47.4 (30); 47 (20, 73)	48.4 (29.8); 48 (21, 74)	33.6 (28.0); 23 (9.2, 52.7)	0.0004
ExFW3t	3239 (418); 3233 (2992, 3498)	3247 (411); 3235 (2999, 3503)	3135 (497.3); 3176 (2877, 3465)	0.1255
%ExFW3t	97 (13.6); 96.6 (87.4, 105.5)	97.52 (13.5); 97.2 (88.4, 105.6)	89.9 (13); 90 (81.1, 97.6)	0.0003
Interval of examination–delivery (days)	6.4 (4); 6 (3, 9)	6.5 (3.9); 6 (3, 9)	5.2 (4.5); 4 (2, 8)	0.0113
Birth weight (grams)	3213 (531); 3250 (2900, 3598)	3239 (516); 3280 (2928, 3600)	2852 (599); 2920 (2471, 3288)	<0.0001
Birth weight centile (local)	39.4 (31.1); 34 (10, 66)	40.6 (31.05); 36 (11, 68)	21.75 (26.12); 11 (3, 35.2)	<0.0001
	**N (%)**	**N (%)**	**N (%)**	
Nulliparity	411 (52.9)	378 (52.1)	33 (63.5)	0.1498
Smoking	110 (14.1)	105 (14.5)	5 (9.6)	0.4135
Male sex	389 (50.1)	359 (49.5)	30 (57.7)	0.0066
Type of labor onset				
Induction of labor	393 (50.6)	348 (48)	45 (86.5)	<0.0001
Spontaneous onset of labor	384 (49.4)	377 (52)	7 (13.4)	<0.0001
Apgar < 7 at 5 min	4 (0.5)	3 (0.4)	1 (1.9)	0.2424
Arterial pH < 7.10	18 (2.3)	12 (1.6)	6 (11.5)	0.0007
Mode of birth				
Cesarean section (failure to progress)	92 (11.8)	92 (12.7)	0 (0)	0.0027
Cesarean section (abnormal CTG)	52 (6.7)	0 (0)	52 (100)	<0.0001
Assisted vaginal delivery	174 (22.4)	174 (24)	0 (0)	<0.0001
Spontaneous vaginal delivery	459 (59.1)	459 (63.3)	0 (0)	<0.0001
Neonatal transfer				
Maternal ward	738 (95%)	698 (96.3)	40 (77)	<0.0001
Neonatal ward	38 (4.9)	27 (3.7)	11 (23)	<0.0001
Neonatal intensive care unit (NICU)	1 (0.1)	0 (0)	1 (0)	0.0669

**Notes**: * Mann–Whitney U test, ExFW3t: expected weight achieved in the 3rd trimester, %ExFW3t: percentage of expected weight achieved in the 3rd trimester, UA PI MoM: umbilical artery pulsatility index multiples of the median, MCA PI: middle cerebral artery pulsatility index multiples of the median, CPR: cerebroplacental ratio, CTG: cardiotocogram (fetal monitoring), SD: standard deviation, 3rd trim: third trimester, IFC: intrapartum fetal compromise, IQR: interquartile range.

**Table 2:** Comparison of the different parameters expressed as univariable models for the prediction of intrapartum fetal compromise. The best parameter, according to the AUC and AIC, was the MCA PI MoM.

Univariable Model	Estimate	OR	95% CI	*p*-Value
**A—maternal parameters**				
**Maternal age**				
Maternal age	0.04212	1.04301	[0.9848, 1.1047]	0.1507
Intercept	−4.02707			
DR. = 4% for a FPR of 5% and 10% for a FPR of 10%, AUC = 0.56, 95% CI [0.49, 0.63], *p* = 0.1566, AIC = 383.6				
**Nulliparity**				
Nulliparity	0.46650	1.5944	[0.8901, 2.8561]	0.1168
Intercept	−2.90489			
DR = 0% for a FPR of 5% and 0% for a FPR of 10%, AUC = 0.56, 95% CI [0.48, 0.64], *p* = 0.1722, AIC = 383.2				
**Maternal height**				
Maternal height	−0.07083	0.9316	[0.8894, 0.9758]	0.0027
Intercept	8.80824			
DR = 17% for a FPR of 5% and 25% for a FPR of 10%, AUC = 0.61, 95% CI [0.52, 0.70], *p* = 0.0087, AIC = 376.6				
**Maternal weight**				
Maternal weight	0.0049	1.0049	[0.9818, 1.0285]	0.6818
Intercept	−2.9392			
DR = 0% for a FPR of 5% and 0% for a FPR of 10%, AUC = 0.51, 95% CI [0.42, 0.60], *p* = 0.7613, AIC = 385.5				
**Smoking**				
Smoking	−0.4649	0.62817	[0.2442, 1.6160]	0.3348
Intercept	−2.5796			
DR = 0% for a FPR of 5% and 0% for a FPR of 10%, AUC = 0.52, 95% CI [0.44, 0.60], *p* = 0.5573, AIC = 384.7				
**B—fetal parameters**				
**B1—fetal sex**				
**Fetal sex**				
Fetal sex	0.3295	1.390	[0.7869, 2.4561]	0.2565
Intercept	−2.812			
DR = 0% for a FPR of 5% and 0% for a FPR of 10%, AUC = 0.54, 95% CI [0.46, 0.62], *p* = NS, AIC = 384.4				
**B2—fetal hemodynamics (fetal Doppler)**				
**MCA PI MoM**				
MCA PI MoM	−3.9666	0.0189	[0.0041, 0.0863]	<0.0001
Intercept	0.8939			
DR = 27% for a FPR of 5% and 33% for a FPR of 10%, AUC = 0.71, 95% CI [0.63, 0.79], *p* < 0.0001, AIC = 355.3				
**UA PI MoM**				
UA PI MoM	1.40944	4.09368	1.51728, 11.0449	0.0054
Intercept	−4.24007			
DR = 13.5% for a FPR of 5% and 19.2% for a FPR of 10%, AUC = 0.58, 95% CI [0.50, 0.67], *p* = 0.046, AIC = 378.5				
**CPR MoM**				
CPR MoM	−2.874	0.0563	[0.0181, 0.1756]	<0.0001
Intercept	0.0514			
DR = 19% for a FPR of 5% and 38% for a FPR of 10%, AUC = 0.70, 95% CI [0.62, 0.78], *p* < 0.0001, AIC = 357.4				
**B3—fetal weight centiles**				
**EFW centiles (local population)**				
EFW centiles (local population)	−0.01851	0.9817	[0.9717, 0.9916]	0.0003
Intercept	−1.950			
DR = 11% for a FPR of 5% and 23% for a FPR of 10%, AUC = 0.66, 95% CI [0.58, 0.73], *p* = 0.0001, AIC = 371.1				
**EFW centiles (Intergrowth 21st)**				
EFW centiles (Intergrowth 21st)	−0.0178	0.9824	[0.9722, 0.9926]	0.0007
Intercept	−1.9094			
DR = 10% for a FPR of 5% and 19% for a FPR of 10%, AUC = 0.64, 95% CI [0.57, 0.72], *p* = 0.0004, AIC = 373.3				
**B4—Percentage of expected weight achieved at the third trimester scan**				
**Percentage of expected weight achieved at third trimester**				
%ExFW3t	−0.04431	0.9567	[0.9353, 0.9784]	0.0001
Intercept	1.515			
DR = 15% for a FPR of 5% and 25% for a FPR of 10%, AUC = 0.66, 95% CI [0.58, 0.73], *p* = 0.0001, AIC = 369.8				

**Notes**: MCA PI MoM: middle cerebral artery pulsatility index multiples of the median, EFW: estimated fetal weight (Hadlock), %ExFW3t: % expected weight achieved in the third trimester, OR: odds ratio, 95% CI: 95% confidence intervals, AUC: area under the curve, AIC: Akaike Information Criteria, DR: detection rate, FPR: false positive rate.

**Table 3:** Comparison of the different multivariable models for predicting intrapartum fetal compromise that included MCA PI MoM. According to the AUC and AIC, the best models were models 3 and 4, which combined MCA PI MoM + EFW centiles (local) or %ExFW3t + maternal height.

Multivariable Model	Estimate	OR	95% CI	*p*-Value
**Model 1: MCA PI MoM + EFW centile (local)**				
MCA PI MoM	−3.555	0.0286	[0.0058, 0.1271]	<0.0001
EFW centile (local)	−0.01402	0.9861	[0.9755, 0.9960]	<0.0077
Intercept	1.054			
DR = 23% for a FPR of 5% and 29% for a FPR of 10%, AUC = 0.74, 95% CI [0.67, 0.81], *p* < 0.0001, AIC = 350				
**Model 2: MCA PI MoM + %ExFW3t**				
MCA PI MoM	−3.491	0.0305	[0.0062, 0.1300]	<0.0001
% EW3t	−0.0331	0.9675	[0.9447, 0.9899]	0.0008
Intercept	3.578			
DR = 25% for a FPR of 5% and 38% for a FPR of 10%, AUC = 0.73, 95% CI [0.66, 0.81], *p* < 0.0001, AIC = 349				
**Model 3: MCA PI MoM + EFW centile (local) + maternal height**				
MCA PI MoM	−3.556	0.0285	[0.0058, 0.1259]	<0.0001
EFW centile (local)	−0.0128	0.9872	[0.9766, 0.9972]	0.0152
Maternal height	−0.0587	0.9430	[0.8993, 0.9878]	0.0139
Intercept	10.5			
DR = 30% for a FPR of 5% and 40% for a FPR of 10%, AUC = 0.75, 95% CI [0.68, 0.82], *p* < 0.0001, AIC = 345				
**Model 4: MCA PI MoM + %ExFW3t + maternal height**				
MCA PI MoM	−3.533	0.02921	[0.0060, 0.1271]	<0.0001
%ExFW3t	−0.02645	0.9739	[0.9536, 0.9938]	0.0011
Maternal height	−0.06240	0.9395	[0.8959, 0.9843]	0.0086
Intercept	13.32			
DR = 27% for a FPR of 5% and 48% for a FPR of 10%, AUC = 0.75, 95% CI [0.67, 0.82], *p* < 0.0001, AIC = 345				

**Notes:** MCA PI MoM: middle cerebral artery pulsatility index multiples of the median, EFW: estimated fetal weight (Hadlock), % EW3t: % expected weight achieved in the third trimester. OR: odds ratio, 95% CI: 95% confidence intervals, AUC: area under the curve, AIC: Akaike Information Criteria, DR: detection rate, FPR: false positive rate.

**Table 4:** Comparison of the different multivariable models used for predicting intrapartum fetal compromise that included CPR MoM. According to the AUC and AIC, the best model was model 8, which combined CPR MoM + %ExFW3t + maternal height.

Multivariable Model	Estimate	OR	95% CI	*p*-Value
**Model 5: CPR MoM + EFW centile (local)**				
CPR MoM	−2.4357	0.0875	[0.0266, 0.2873]	<0.0001
EFW centile (local)	−0.0113	0.9888	[0.9783, 0.9994]	<0.0382
Intercept	−0.0287			
DR = 23% for a FPR of 5% and 35% for a FPR of 10%, AUC = 0.72, 95% CI [0.64, 0.79], *p* < 0.0001, AIC = 355				
**Model 6: CPR MoM + %ExFW3t**				
CPR MoM	−2.3532	0.0950	[0.0295, 0.3064]	<0.0001
% EW3t	−0.0369	0.9637	[0.9409, 0.9871]	0.0025
Intercept	2.8684			
DR = 23% for a FPR of 5% and 35% for a FPR of 10%, AUC = 0.73, 95% CI [0.65, 0.80], *p* < 0.0001, AIC = 353				
**Model 7: CPR MoM + EFW centile (local) + maternal height**				
CPR MoM	−2.4285	0.0882	[0.0270, 0.2882]	<0.0001
EFW centile (local)	−0.0098	0.9902	[0.0054, 0.0725]	0.0725
Maternal height	−0.0564	0.9452	[0.0238, 0.0177]	0.0177
Intercept	9.0201			
DR = 27% for a FPR of 5% and 38% for a FPR of 10%, AUC = 0.74, 95% CI [0.66, 0.81], *p* < 0.0001, AIC = 351				
**Model 8: CPR MoM + %ExFW3t + Maternal height**				
CPR MoM	−2.3992	0.0908	[0.0283, 0.2914]	<0.0001
%ExFW3t	−0.0275	0.9728	[0.9504, 0.9957]	0.0204
Maternal height	−0.0574	0.9442	[0.9014, 0.9891]	0.0154
Intercept	11.3775			
DR = 25% for a FPR of 5% and 40% for a FPR of 10%, AUC = 0.76, 95% CI [0.69, 0.83], *p* < 0.0001, AIC = 345.5				

**Notes**: EFW: estimated fetal weight (Hadlock), % EW3t: % expected weight achieved in the third trimester. OR: odds ratio, 95% CI: 95% confidence intervals, AUC: area under the curve, AIC: Akaike Information Criteria, DR: detection rate, FPR: false positive rate.

**Table 5:** Performance of the different univariable and multivariable models, ordered according to the lowest AIC (highest accuracy and reproducibility).

Models	AUC	AIC	DR for a FPR of 5%	DR for a FPR of 10%	*p*-Value
**Univariable models**					
Maternal weight	0.51	385.5	0	0	0.7613
Smoking	0.52	384.7	0	0	0.5573
Fetal sex	0.54	384.4	0	0	0.3243
Maternal age	0.56	383.6	4	10	0.1566
Nulliparity	0.56	383.2	0	0	0.1722
UA PI MoM	0.58	378.5	13.5	19.2	0.0460
Maternal height	0.61	376.6	17	25	0.0087
EFW centiles (EFWc), 21st intergrowth	0.64	373.3	10	19	0.0007
EFW centiles (EFWc), local population	0.66	371.1	11	23	0.0003
Percentage of expected weight achieved at third trimester (%ExFW3t)	0.66	369.8	15	25	0.0001
CPR MoM	0.70	357.4	19	38	<0.0001
MCA PI MoM	0.71	355.3	27	33	<0.0001
**Multivariable models**					
Model 5: CPR MoM + EFWc, local population	0.72	355	23	35	< 0.0001
Model 6: CPR MoM + %ExFW3t	0.73	353	23	35	< 0.0001
Model 7: CPR MoM + EFWc, local population + maternal height	0.74	351	27	38	< 0.0001
Model 1: MCA PI MoM + EFWc, local population	0.74	350	23	29	<0.0001
Model 2: MCA PI MoM + %ExFW3t	0.73	349	25	38	<0.0001
Model 8: CPR MoM + %ExFW3t + maternal height	0.76	345.5	25	40	< 0.0001
Model 3: MCA PI MoM + EFWc, local population + maternal height	0.75	345	30	40	<0.0001
Model 4: MCA PI MoM + %ExFW3t + maternal height	0.75	345	27	48	<0.0001

**Notes**: MCA PI MoM: middle cerebral artery pulsatility index multiples of the median, EFW: estimated fetal weight (Hadlock), %ExFW3t: % expected weight achieved in the third trimester, AUC: area under the curve, AIC: Akaike Information Criteria, DR: detection rate, FPR: false positive rate.

## Data Availability

Data are available upon request from the authors.

## Abbreviations

GA	Gestational age.
IFC	Intrapartum fetal compromise.
EFW	Estimated fetal weight.
CPR	Cerebroplacental ratio.
UA	Umbilical artery.
MCA	Middle cerebral artery.
MoM	Multiples of the median.
FGR	Fetal growth restriction.
PI	Pulsatility index.
BDP	Biparietal diameter.
HC	Head circumference.
AC	Abdominal circumference.
FL	Femur length.
ExFW3t	Expected weight at the 3rd trimester.
%ExFW3t	Percentage of ExFW at the 3rd trimester.
AIC	Akaike information criteria.
DR	Detection rate.
FPR	False positive rate.
AUC	Area under the curve.
